# IUC23424-80 Comparative analysis of wallace versus bricker anastomosis in radical cystectomy: a prospective study of 50 patients

**DOI:** 10.1093/oncolo/oyaf248.025

**Published:** 2025-08-29

**Authors:** O V Potdar

**Affiliations:** Mumbai, India

## Abstract

**Background:**

To compare the outcomes of Wallace and Bricker anastomosis techniques in urinary diversion following radical cystectomy for bladder cancer.

**Methods:**

A prospective study was conducted on 50 patients undergoing radical cystectomy with ileal conduit diversion. Patients were randomized into 2 groups: Wallace anastomosis (*n* = 25) and Bricker anastomosis (*n* = 25). The primary endpoints included operative time, anastomotic leakage, stricture formation, urinary tract infections (UTI), and long-term renal function.

**Results:**

Operative Time: The mean operative time was significantly lower in the Bricker group (*P* < .05). Anastomotic Leakage: Wallace group showed a slightly higher, but statistically insignificant, incidence of early leakage (12% vs 8%). Stricture Formation: Stricture rates were comparable between the groups (Wallace: 8%, Bricker: 4%). UTI Rates: Postoperative UTIs were more frequent in the Bricker group (36% vs 28%). Renal Function: No significant long-term deterioration in renal function was observed in either group.

**Conclusions:**

Both Wallace and Bricker anastomosis techniques are viable options for ileal conduit urinary diversion. Wallace anastomosis may have a slightly higher leakage rate, but it does not significantly affect long-term outcomes. The Bricker technique demonstrated a shorter operative time, making it preferable in high-risk patients. Further large-scale studies are required to establish definitive recommendations.

